# A-to-I RNA editing impairs miR-376b-3p repression of *RYBP* in skeletal muscle satellite cells

**DOI:** 10.1016/j.jbc.2025.111006

**Published:** 2025-12-05

**Authors:** Xiaoli Xu, Chengqi Wei, Peijie Zeng, Siyuan Zhan, Dinghui Dai, Li Li, Hongping Zhang

**Affiliations:** Farm Animal Genetic Resources Exploration Innovation Key Laboratory of Sichuan Province, College of Animal Science and Technology, Sichuan Agricultural University, Chengdu, China

**Keywords:** A-to-I editing, miR-376b-3p, *RYBP*, MuSCs, proliferation, differentiation

## Abstract

Adenosine-to-inosine RNA editing can affect miRNA activity, but its role in skeletal muscle development remains unclear. Here, we investigated miR-376b-3p in goat skeletal muscle satellite cells (MuSCs), which undergo adenosine deaminase acting on RNA 1–mediated editing at the sixth nucleotide of its seed sequence. Although both isoforms were detected, the unedited miR-376b-3p (miR-WT) predominated over the edited form (miR-E) during skeletal muscle development and MuSC differentiation. Functional assays revealed that miR-WT, but not the miR-E type, enhanced MuSC proliferation and differentiation by upregulating *Pax7*, *PCNA*, *MyoD*, *MyoG*, and *MyHC*, and promoting myotube formation. Furthermore, we identified Ring1 and YY1 binding protein (RYBP), a repressor of myogenesis, as a direct target of miR-WT. Overexpression of *RYBP* inhibited MuSC differentiation, whereas miR-WT relieved this repression through direct binding to the *RYBP* 3′UTR. In contrast, miR-E failed to target *RYBP* and lacked promyogenic activity. These findings demonstrate that adenosine-to-inosine editing attenuates the function of miR-376b-3p, highlighting its role as a post-transcriptional regulator of skeletal muscle development.

Mammalian skeletal muscle is the largest tissue in the body, accounting for ∼40% of body weight, and plays essential roles in locomotion, thermogenesis, and metabolism. In domestic animals, it also represents the primary source of meat, making its growth directly relevant to economic value. Postnatal muscle growth primarily depends on the hypertrophy of existing fibers and the activity of skeletal muscle satellite cells (MuSCs), a stem cell population responsible for fiber hypertrophy, repair, and maintaining the stem cell pool (1). MuSCs reside in a quiescent state beneath the basal lamina. Upon mechanical stimuli or injury, they become activated, re-enter the cell cycle, proliferate, and subsequently differentiate into myocytes that fuse with existing fibers to support muscle growth and regeneration (2, 3). The transition of MuSCs from quiescence to activation is coordinated by a multilayered regulatory network involving transcription factors (4), epigenetic regulators (5), structural proteins (6), and noncoding RNAs (7). Among these, miRNAs, small noncoding RNAs that post-transcriptionally repress gene expression, have emerged as crucial regulators of myogenesis. Both muscle-specific miRNAs (*e*.*g*., miR-1, miR-133, and miR-206) and broadly expressed miRNAs (*e*.*g*., miR-27b, miR-26a, and miR-193b-3p) contribute to proliferation, differentiation, and fusion of myogenic cells by targeting key myogenic regulators (8, 9, 10).

Given the role of miRNAs in regulating myogenic cell function, a crucial but underexplored aspect is how post-transcriptional modifications of miRNAs, particularly adenosine-to-inosine (A-to-I) RNA editing, influence their activity. A-to-I editing, catalyzed by adenosine deaminases acting on RNA (ADARs), converts adenosine to inosine in double-stranded RNA regions. The ADAR family comprises three members (ADAR1, ADAR2, and ADAR3), among which ADAR1 is indispensable for embryonic viability, as its knockout in mice results in extensive cell death and embryonic lethality (11, 12). In skeletal muscle, accumulating evidence indicates that RNA editing activity is developmentally regulated. For instance, a developmental atlas of the RNA editome in pig showed that both the frequency and overall level of RNA editing gradually decline during muscle development, accompanied by reduced ADAR1 mRNA and protein expression (13). Similarly, editing events are more abundant during fetal stages than postnatally in goats (14). However, RNA editing levels are lower in embryonic muscle compared with adult muscle in yak, suggesting species-specific dynamics (15). Collectively, these studies indicate that RNA editing is dynamically modulated during skeletal muscle development. At the cellular level, *ADAR1* expression and global A-to-I editing activity increase during early myoblast differentiation (16, 17, 18, 19), implying that RNA editing may actively contribute to myogenic progression and the regulation of MuSC function.

A-to-I editing can occur in both coding and noncoding RNAs, including pri-miRNAs and mature miRNAs (20). In pri-miRNAs, editing can suppress processing by Drosha, as demonstrated for pri–miR-142, whose edited form is degraded by Tudor-SN, leading to altered levels of mature miRNA (21). In mature miRNAs, A-to-I editing can generate sequence variants with altered target specificity, leading to potential changes in the set of genes regulated by the edited miRNAs and affecting gene expression (22). Recent studies further highlight that A-to-I editing dynamically regulates miRNA activity in a context-dependent manner. Editing levels can vary across developmental stages or pathological states, shifting the balance between edited and unedited isoforms and modulating their biological effects (23). In cancer, for example, edited isoforms of miR-1304-3p, miR-154-p13-5p, and miR-379-5p acquire altered functions by redirecting targets, such as *Wnt5a*/*ROR2*, *LIX1L*, or *CD97*, thereby influencing proliferation, apoptosis, migration, and therapeutic response (24, 25, 26). Moreover, editing itself can be regulated by noncoding RNAs, as seen in the circNEIL3–miR-432-5p–ADAR1 feedback loop in pancreatic ductal adenocarcinoma (27). Collectively, these findings establish RNA editing as a versatile mechanism of post-transcriptional control, thereby expanding the functional diversity of miRNA. Among the miRNAs potentially regulated by A-to-I editing, the miR-379/410 cluster, the largest miRNA cluster in mammals, is of particular interest. Members of this cluster are widely expressed during embryonic and postnatal development, and many harbor conserved editing sites in their seed sequences (28, 29). miR-376b-3p, a member of this cluster, has been reported to undergo A-to-I editing in mice, where ADAR1 and ADAR2 coordinately regulate its maturation and target specificity in the brain (29). Transcriptome sequencing of the longissimus dorsi (LD) muscle in Dazu Black goats revealed that miR-376b-3p is highly expressed at embryonic day 75 compared with postnatal day 1, suggesting a potential role in myogenesis (30). However, whether this editing occurs in skeletal muscle and how it affects the function of MuSCs remains unknown.

In this study, we systematically investigated the impact of A-to-I editing on miR-376b-3p in goat MuSCs. By profiling edited and unedited isoforms and conducting gain-of-function experiments, we demonstrate that RNA editing modifies the target landscape of miR-376b-3p and attenuates its promyogenic activity, thereby influencing MuSC proliferation and differentiation. These findings reveal an underappreciated mechanism of miRNA regulation in skeletal muscle, providing mechanistic insights with potential implications for enhancing livestock muscle growth.

## Results

### A-to-I editing of miR-376b-3p and its expression in goat MuSCs

**Figure 1:**
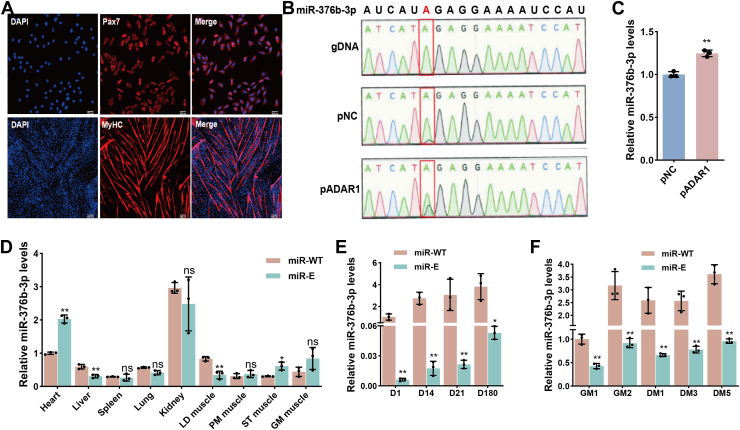
**Expression patterns of miR-376b-3p and its edited form in goat skeletal muscle satellite cells (MuSCs)**. *A*, immunofluorescence identification of MuSCs using Pax7 (a MuSC marker) and MyHC (a marker of differentiated myotubes). *B*, Sanger sequencing analysis of the miR-376b-3p sequence following transfection with *ADAR1* overexpression plasmid (pADAR1) or negative control vector (pNC), revealing A-to-I RNA editing events. *C*, RT–qPCR analysis of edited miR-376b-3p expression following transfection with pADAR1 or pNC, and the data were evaluated using the *t* test. Data are represented as the mean ± SD, n = 3 independent experiments. *D*, tissue-specific expression profiles of miR-376b-3p and its edited form in goat tissues, and the data were evaluated using the *t* test. Data are represented as the mean ± SD, n = 3 independent experiments. *E*, the expression patterns of miR-376b-3p and its edited form at postnatal days 1 (D1), 14 (D14), 21 (D21), and 180 (D180) during skeletal muscle development, and the data were evaluated using the *t* test. Data are represented as the mean ± SD, n = 3 independent experiments. *F*, dynamic expression patterns of miR-376b-3p and its edited form during MuSC proliferation and differentiation, and the data were evaluated using the *t* test. GM1 and GM2 cells were cultured in proliferation medium for 1 and 2 days, respectively, whereas DM1, DM3, and DM5 cells were cultured in myogenic differentiation medium for 1, 3, and 5 days, respectively. Data are represented as the mean ± SD, n = 3 independent experiments. *Asterisks* indicate statistical significance: ∗ for *p* < 0.05, ∗∗ for *p* < 0.01, and “ns” for nonsignificant differences. A-to-I, adenosine-to-inosine; DM, differentiation medium; GM, growth medium; qPCR, quantitative PCR.

To investigate the functional role of miR-376b-3p, we first isolated skeletal MuSCs from the LD muscle of goats and verified their identity by immunofluorescence staining for Pax7 and MyHC (Fig. 1*A*). Previous studies have shown that the sixth nucleotide in the seed sequence of miR-376b-3p is prone to RNA A-to-I editing, recognized as an A-to-G substitution, a modification primarily catalyzed by ADAR1, which can alter miRNA function (29, 31). To assess the editing status of goat miR-376b-3p, we overexpressed ADAR1 in MuSCs and examined the editing level using Sanger sequencing. The G peak at the sixth nucleotide position of miR-376b-3p was barely detectable in cells lacking ADAR1 overexpression but was markedly increased when ADAR1 was overexpressed (Fig. 1*B*). Consistently, RT–quantitative PCR (qPCR) analysis revealed that ADAR1 overexpression significantly increased the expression level of the edited form (miR-E) (Fig. 1*C*). These results indicate that A-to-I editing occurs in goat miR-376b-3p, but the unedited form (miR-WT) predominates in MuSCs.

We next compared the tissue distribution patterns of miR-WT and miR-E across various tissues. miR-E levels were significantly higher than miR-WT in the heart and semitendinosus muscle, whereas miR-WT levels were higher in the liver and LD muscle (Fig. 1*D*). To determine whether miR-376b-3p editing is developmentally regulated, we examined the expression of the miR-WT and miR-E forms across postnatal muscle development and in cultured MuSCs undergoing proliferation and differentiation. Both miR-WT and miR-E showed a general increasing trend during postnatal development, with miR-WT consistently more abundant than miR-E at all stages, indicating that A-to-I editing activity progressively rises as development advances (Fig. 1*E*). In an *in vitro* MuSC differentiation model, both miR-WT and miR-E levels increased at the late proliferation stage (day 2) and then declined during the early differentiation stages (days 1 and 3), suggesting that A-to-I editing activity generally increases during differentiation. Notably, miR-WT expression consistently remained significantly higher than miR-E throughout the differentiation process (Fig. 1*F*). Collectively, these findings demonstrate that A-to-I editing of miR-376b-3p is developmentally regulated and may play a role in fine-tuning myoblast proliferation and differentiation.

### Unedited miR-376b-3p enhances MuSC proliferation

**Figure 2:**
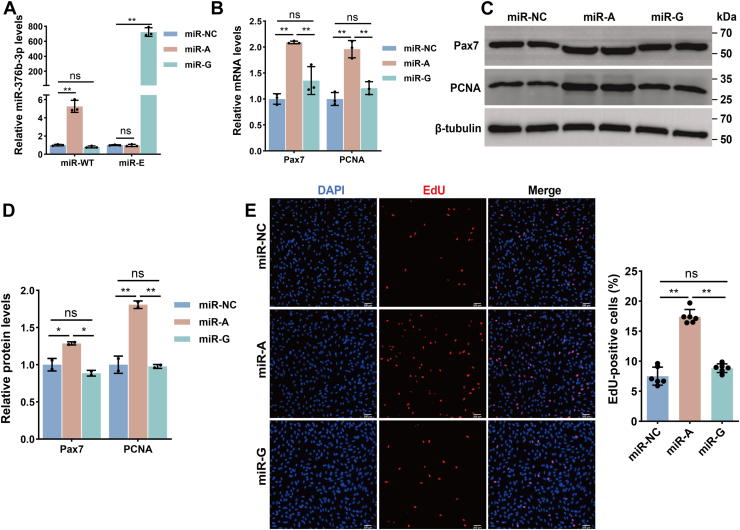
**Effects of WT and edited miR-376b-3p on the proliferation of goat MuSCs**. *A*, expression of WT miR-376b-3p (miR-WT) and its edited form (miR-E) in MuSCs following transfection with miR-376b-3p mimic (miR-A) and its edited form (miR-G), or negative control (miR-NC), and the data were evaluated using the one-way ANOVA. Data are represented as the mean ± SD, n = 3 independent experiments. *B*, mRNA expression of proliferation-related genes, *Pax7* and *PCNA*, following transfection with miR-A, miR-G, or miR-NC in MuSCs, and the data were evaluated using the one-way ANOVA. Data are represented as the mean ± SD, n = 3 independent experiments. *C* and *D*, Western blot analysis of Pax7 and PCNA protein levels in MuSCs transfected with miR-A, miR-G, or miR-NC, and the data were evaluated using the one-way ANOVA. Data are represented as the mean ± SD, n = 2 independent experiments. *E*, EdU assay to assess MuSC proliferation following transfection with miR-A, miR-G, or miR-NC. The percentage of EdU-positive cells is shown on the *right*, and the data were evaluated using the one-way ANOVA. Data are represented as the mean ± SD, n = 6 independent experiments. *Asterisks* indicate statistical significance: ∗ for *p* < 0.05, ∗∗ for *p* < 0.01, and “ns” for nonsignificant differences. EdU, 5-ethynyl-2′-deoxyuridine; MuSC, skeletal muscle satellite cell.

To investigate the functional roles between the unedited and edited forms of miR-376b-3p in regulating goat MuSC proliferation, we transfected MuSCs with mimics of the miR-WT (miR-A), the miR-E (miR-G), or a negative control (miR-NC) at growth medium day 1. Transfection efficiency was confirmed using locked nucleic acid–modified primers, which successfully detected the expression of both miR-WT and miR-E (Fig. 2*A*). Given that *Pax7* and *PCNA* are well-recognized markers of cell proliferation (32, 33), we next examined their expression following transfection to evaluate proliferative capacity. We found that overexpression of miR-A significantly increased the mRNA and protein levels of *Pax7* and *PCNA* compared with the miR-NC group, whereas miR-G overexpression had no significant effect on the expression of these proliferation markers (Fig. 2, *B*–*D*). Consistently, 5-ethynyl-2′-deoxyuridine (EdU) incorporation assays revealed that miR-A markedly increased the proportion of EdU-positive cells relative to both miR-G and miR-NC (Fig. 2*E*). Taken together, these findings indicate that the unedited form of miR-376b-3p promotes MuSC proliferation, whereas the edited form exerts no significant effect.

### Unedited miR-376b-3p promotes MuSC differentiation

**Figure 3:**
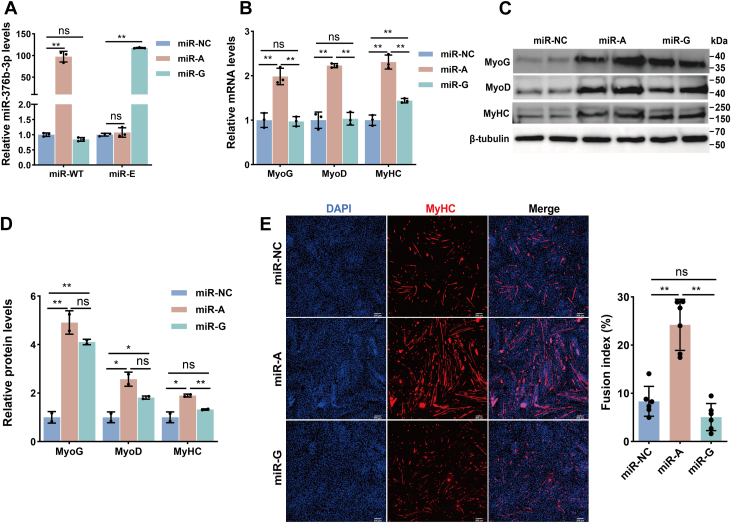
**Effects of WT and edited miR-376b-3p on the differentiation of goat MuSCs**. *A*, expression of miR-WT and miR-E following transfection with miR-A, miR-G, or miR-NC in MuSCs, and the data were evaluated using the one-way ANOVA. Data are represented as the mean ± SD, n = 3 independent experiments. *B*, mRNA expression of differentiation markers *MyoD* (an early myogenic determination factor), *MyoG* (a marker of myoblast differentiation), and *MyHC* (indicative of terminal differentiation and myotube formation) following transfection with miR-A, miR-G, or miR-NC in MuSCs. Data were analyzed using one-way ANOVA and are presented as the mean ± SD (n = 3 independent experiments). *C* and *D*, Western blot analysis of MyoG, MyoD, and MyHC protein levels in MuSCs transfected with miR-A, miR-G, or miR-NC, and the data were evaluated using the one-way ANOVA. Data are represented as the mean ± SD, n = 2 independent experiments. *E*, immunofluorescence staining of MyHC to evaluate the differentiation potential of MuSCs after transfection with miR-A, miR-G, or miR-NC. The fusion index is quantified on the *right*, and the data were evaluated using the one-way ANOVA. Data are represented as the mean ± SD, n = 6 independent experiments. *Asterisks* indicate statistical significance: ∗ for *p* < 0.05, ∗∗ for *p* < 0.01, and “ns” for nonsignificant differences. MuSC, skeletal muscle satellite cell.

To elucidate the distinct roles of unedited and edited miR-376b-3p in MuSC differentiation, we transfected their respective mimics, miR-A, miR-G, and miR-NC into MuSCs at day 1 of differentiation. Transfection efficiency verification confirmed robust overexpression of both miR-A and miR-G (Fig. 3*A*). RT–qPCR analysis demonstrated that miR-A significantly upregulated the mRNA and protein expression of myogenic marker genes *MyoG* and *MyoD* (34), as well as the muscle structural protein *MyHC* (6), compared with miR-NC. In contrast, miR-G exerted no effect on *MyoG* and *MyoD* mRNA levels, which remained unchanged, but enhanced their protein abundance, and increased *MyHC* mRNA without altering its protein level (Fig. 3, *B*–*D*). Immunofluorescence staining of MyHC further corroborated these findings. Quantification of nuclei within multinucleated myotubes revealed that miR-A markedly increased the proportion of nuclei incorporated into myotubes relative to miR-NC, indicative of enhanced myogenic differentiation. Conversely, miR-G showed no significant effect on myotube formation compared with miR-NC (Fig. 3*E*). Taken together, these results indicate that the unedited miR-376b-3p robustly promotes both the transcription and translation of key myogenic factors, thereby enhancing MuSC differentiation, whereas the edited form exhibits a limited regulatory effect.

### Ring1 and YY1 binding protein predicted as a direct target of unedited miR-376b-3p

**Figure 4:**
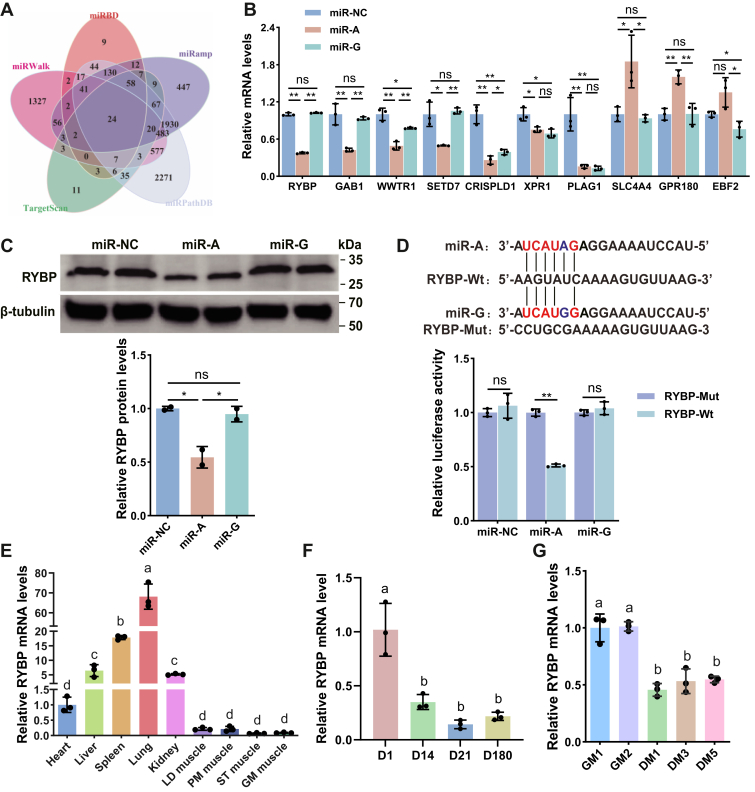
**Screening and validation of miR-376b-3p target genes**. *A*, prediction of potential target genes of miR-376b-3p using five commonly used miRNA target prediction databases. *B*, RT–qPCR validation of randomly selected predicted targets to assess the effects of miR-A, miR-G, or miR-NC in differentiated goat MuSCs, and the data were evaluated using the one-way ANOVA. Data are represented as the mean ± SD, n = 3 independent experiments. *C*, Western blot analysis of RYBP protein expression following transfection with miR-A, miR-G, or miR-NC in differentiated goat MuSCs, and the data were evaluated using the one-way ANOVA. Data are represented as the mean ± SD, n = 2 independent experiments. *D*, dual-luciferase reporter assay validating the effects of miR-A, miR-G, or miR-NC on the WT (RYBP-WT) and mutant (RYBP-Mut) 3′UTR of *RYBP* in differentiated goat MuSCs, and the data were evaluated using the *t* test. Data are represented as the mean ± SD, n = 3 independent experiments. *E*, tissue-specific mRNA expression profile of *RYBP* in goats and the data were evaluated using the one-way ANOVA. Data are represented as the mean ± SD, n = 3 independent experiments. *F*, expression pattern of *RYBP* mRNA during skeletal muscle development at different stages, and the data were evaluated using the one-way ANOVA. Data are represented as the mean ± SD, n = 3 independent experiments. *G*, expression pattern of *RYBP* mRNA during MuSC proliferation and differentiation, and the data were evaluated using the one-way ANOVA. Data are represented as the mean ± SD, n = 3 independent experiments. *Asterisks* indicate statistical significance: ∗ for *p* < 0.05, ∗∗ for *p* < 0.01, and “ns” for nonsignificant differences. qPCR, quantitative PCR; RYBP, Ring1 and YY1 binding protein.

To elucidate the molecular mechanisms by which the unedited and edited forms of miR-376b-3p regulate goat MuSC proliferation and differentiation, we first predicted the potential target genes of miR-376b-3p. Five miRNA target prediction databases (miRDB, TargetScan, miRPathDB, miRmap, and miRWalk) were used, and 24 shared candidate target genes were identified (Fig. 4*A*). To validate these predictions, we selected 10 candidates for RT–qPCR analysis following overexpression of either miR-A or miR-G in goat differentiation MuSCs. Four distinct regulatory patterns emerged: genes suppressed by miR-A but elevated by miR-G (*e*.*g*., *RYBP*, *GAB1*, *SETD7*); genes consistently downregulated by both miR-A and miR-G (*e*.*g*., *WWTR1*, *CRISPLD1*, *XPR1*, *PLAG1*); genes selectively upregulated by miR-A with no effect of miR-G (*e*.*g*., *SLC4A4*, *GPR180*); and genes unaffected by miR-A but repressed by miR-G (*e*.*g*., *EBF2*) (Fig. 4*B*). Among these, Ring1 and YY1 binding protein (RYBP) has been reported as a negative regulator of myogenic differentiation in mice and cattle (35, 36). Consistently, Western blot analysis demonstrated that RYBP protein abundance was markedly reduced by miR-A overexpression in differentiated goat MuSCs, whereas miR-G exerted negligible effects (Fig. 4*C*), supporting *RYBP* as a target of the unedited form. Direct targeting was confirmed by dual-luciferase assays performed in differentiated goat MuSCs, in which miR-A significantly reduced luciferase activity relative to miR-G by binding to the predicted *RYBP* 3′UTR site (Fig. 4*D*). Analysis of endogenous *RYBP* expression revealed relatively low transcript levels in skeletal muscle compared with visceral tissues (Fig. 4*E*). Furthermore, examination of *RYBP* expression across postnatal developmental stages showed a gradual decline as development progressed (Fig. 4*F*). Similarly, *RYBP* expression progressively decreased during goat MuSC proliferation and differentiation, with substantially lower levels observed in differentiating cells compared with proliferating cells (Fig. 4*G*). Notably, the developmental and proliferation–differentiation expression patterns of *RYBP* were inversely correlated with those of both miR-WT and miR-E (Fig. 1*E*). Collectively, these findings identify *RYBP* as a direct and functional target of the unedited miR-376b-3p.

### *RYBP* suppresses MuSC differentiation without affecting proliferation

**Figure 5:**
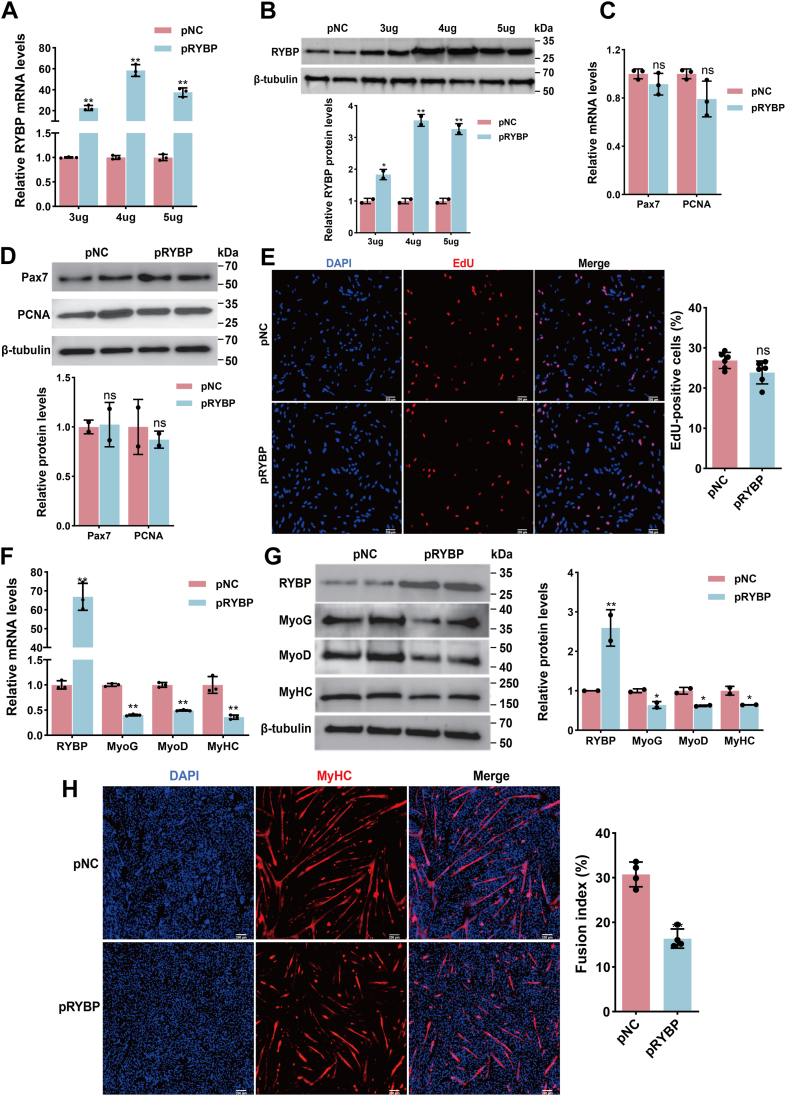
**Functional effects of *RYBP* overexpression on goat MuSC proliferation and differentiation**. *A*, mRNA expression of *RYBP* in MuSCs transfected with different concentrations of *RYBP* overexpression plasmid (pRYBP) or empty vector (pNC), and the data were evaluated using the *t* test. Data are represented as the mean ± SD, n = 3 independent experiments. *B*, protein expression of RYBP following transfection with different concentrations of pRYBP or pNC in MuSCs, and the data were evaluated using the *t* test. Data are represented as the mean ± SD, n = 2 independent experiments. *C*, mRNA expression of proliferation-related genes, *Pax7* and *PCNA*, in MuSCs after overexpression of *RYBP*, and the data were evaluated using the *t* test. Data are represented as the mean ± SD, n = 3 independent experiments. *D*, Western blot analysis of Pax7 and PCNA protein expression following overexpression of *RYBP*, and the data were evaluated using the *t* test. Data are represented as the mean ± SD, n = 2 independent experiments. *E*, EdU incorporation assay to assess the proliferation capacity of MuSCs after overexpression of *RYBP*. The percentage of EdU-positive cells is shown on the *right*, and the data were evaluated using the *t* test. Data are represented as the mean ± SD, n = 6 independent experiments. *F*, mRNA expression of *RYBP* and myogenic differentiation markers in MuSCs after overexpression of *RYBP*, and the data were evaluated using the *t* test. Data are represented as the mean ± SD, n = 3 independent experiments. *G*, protein expression of RYBP and differentiation markers following overexpression of *RYBP*, and the data were evaluated using the *t* test. Data are represented as the mean ± SD, n = 2 independent experiments. *H*, immunofluorescence staining of MyHC to evaluate MuSC differentiation after overexpression of *RYBP*. The fusion index is shown on the *right*, and the data were evaluated using the *t* test. Data are represented as the mean ± SD, n = 4 independent experiments. *Asterisks* indicate statistical significance: ∗ for *p* < 0.05, ∗∗ for *p* < 0.01, and “ns” for nonsignificant differences. EdU, 5-ethynyl-2′-deoxyuridine; MuSC, skeletal muscle satellite cell; RYBP, Ring1 and YY1 binding protein.

To investigate the role of *RYBP* in MuSC proliferation and differentiation, we first optimized the transfection concentration of the *RYBP* plasmid (pRYBP). Overexpression of *RYBP* significantly increased its mRNA and protein levels at 3 μg, 4 μg, and 5 μg transfection doses compared with the control plasmid (pNC), with 4 μg identified as the optimal concentration (Fig. 5, *A* and *B*). Notably, *RYBP* overexpression showed a minor downward trend in *Pax7* and *PCNA* mRNA, as well as *PCNA* protein levels (Fig. 5, *C* and *D*), but these changes were not statistically significant. Consistently, EdU assays revealed no significant change in the proportion of proliferating cells (Fig. 5*E*), indicating that *RYBP* does not substantially impact MuSC proliferation.

We next assessed the effect of *RYBP* during MuSC differentiation. Overexpression of *RYBP* in differentiating MuSCs significantly elevated its mRNA and protein levels. Strikingly, *RYBP* overexpression markedly suppressed the mRNA and protein expression of *MyoG*, *MyoD*, and *MyHC* (Fig. 5, *F* and *G*). Immunofluorescence staining of MyHC further demonstrated that *RYBP* overexpression substantially inhibited myotube formation (Fig. 5*H*). Collectively, these results indicate that *RYBP* acts as a negative regulator of MuSC differentiation, while having minimal impact on proliferation.

### Unedited miR-376b-3p promotes MuSC differentiation by targeting *RYBP*

**Figure 6:**
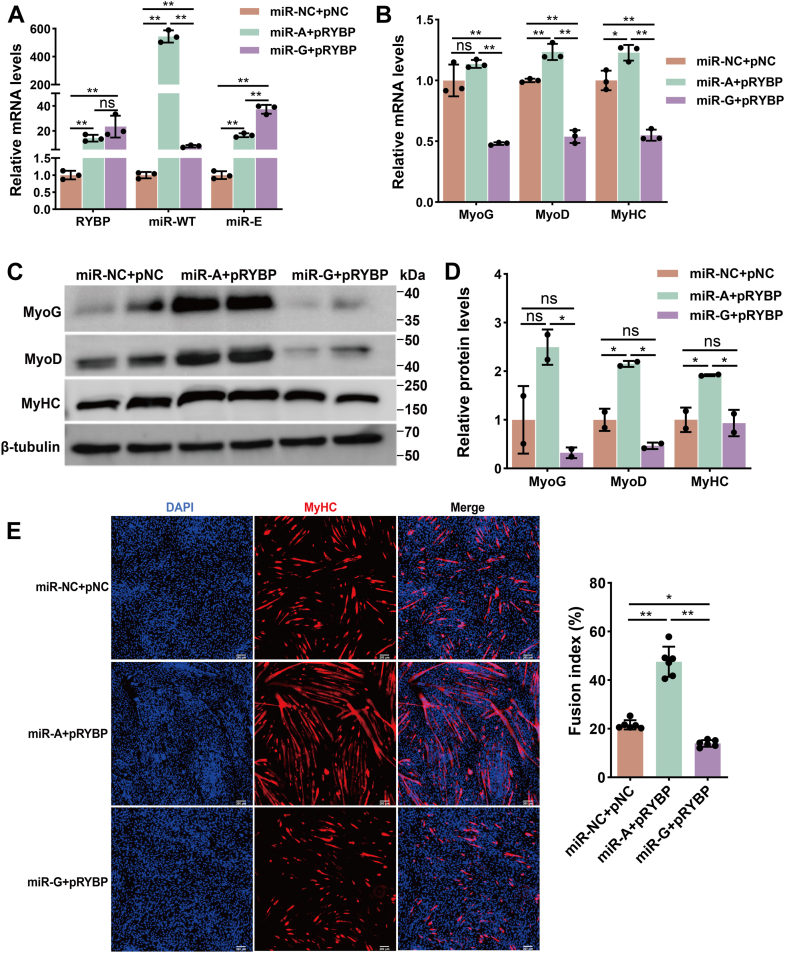
**Effects of miR-376b-3p and its edited form and *RYBP* overexpression on MuSC differentiation**. *A*, quantification of cotransfection efficiency of *RYBP*, miR-WT, and miR-E at the differentiation stage of MuSCs, and the data were evaluated using the one-way ANOVA. Data are represented as the mean ± SD, n = 3 independent experiments. *B*, mRNA expression of myogenic differentiation markers following cotransfection of pRYBP with miR-A or miR-G, and the data were evaluated using the one-way ANOVA. Data are represented as the mean ± SD, n = 3 independent experiments. *C* and *D*, Western blot analysis of MyoG, MyoD, and MyHC protein levels in MuSCs after cotransfection with pRYBP and miR-A or miR-G, and the data were evaluated using the one-way ANOVA. Data are represented as the mean ± SD, n = 2 independent experiments. *E*, immunofluorescence staining of MyHC to assess myotube formation after cotransfection with pRYBP and miR-A or miR-G. The fusion index is shown on the *right*, and the data were evaluated using the one-way ANOVA. Data are represented as the mean ± SD, n = 6 independent experiments. *Asterisks* indicate statistical significance: ∗ for *p* < 0.05, ∗∗ for *p* < 0.01, and “ns” for nonsignificant differences. MuSC, skeletal muscle satellite cell; pRYBP, RYBP plasmid; RYBP, Ring1 and YY1 binding protein.

To further elucidate the interplay between unedited/edited miR-376b-3p and *RYBP* during MuSC differentiation, we first examined the expression of miR-WT, miR-E, and *RYBP* following cotransfection. The results confirmed robust overexpression of each component in their respective groups, indicating successful transfection and effective experimental combinations (Fig. 6*A*). Subsequently, RT–qPCR analysis revealed that cotransfection of miR-A with pRYBP significantly upregulated the mRNA and protein levels of *MyoD* and *MyHC* compared with the control group (miR-NC + pNC) (Fig. 6, *B*–*D*). In contrast, the miR-G and pRYBP cotransfection markedly reduced the mRNA expression of *MyoG*, *MyoD*, and *MyHC* (Fig. 6*B*), while showing no significant changes at the protein level (Fig. 6, *C* and *D*). Consistently, MyHC immunofluorescence staining demonstrated that miR-A and pRYBP cotransfection resulted in the formation of hypertrophic myotubes with a higher fusion index relative to the control, whereas cotransfection of miR-G with pRYBP produced fewer and thinner myotubes with a reduced fusion index (Fig. 6*E*). Together, these findings demonstrate that unedited miR-376b-3p promotes MuSC differentiation by directly targeting *RYBP*, thereby relieving its inhibitory effect on myogenesis. In contrast, the edited form fails to bind *RYBP* and consequently exerts little influence on the differentiation process.

## Discussion

Skeletal muscle development is governed by complex transcriptional and post-transcriptional regulatory networks that determine myogenic lineage commitment and differentiation. Among these, miRNAs function as pivotal post-transcriptional regulators by modulating key transcription factors involved in myogenesis (37), integrating signaling pathways, such as the Trib2–mTOR–S6K cascade (38), and cooperating with chromatin-associated epigenetic regulators to regulate muscle development (39). In this study, we demonstrate that unedited miR-376b-3p promotes myogenic differentiation in goat MuSCs by directly targeting *RYBP*, whereas A-to-I editing abolishes this interaction, functioning as a molecular switch that modulates the miRNA’s regulatory activity.

miRNAs regulate muscle development, primarily through complementary binding of their seed sequences to the 3′UTRs of target mRNAs (40). The alteration in the miRNA sequence, particularly within the seed region, can reshape its target spectrum and thereby redefine its biological functions. In addition to canonical mRNA targeting, miRNAs can also interact with RNA-binding proteins and participate in competing endogenous RNA regulatory networks, in which diverse RNA species, such as long noncoding RNAs, circular RNAs, and mRNAs, compete for miRNA binding to modulate gene expression (41). RNA editing, especially A-to-I conversion catalyzed by ADAR enzymes, represents a crucial post-transcriptional modification that not only occurs in coding transcripts but also in miRNA precursors and mature sequences (20, 42). Notably, global RNA editing levels can vary substantially across developmental and disease contexts even when ADAR expression remains relatively stable (43), indicating that editing activity is governed by regulatory mechanisms beyond enzyme abundance. Here, we identified a conserved A-to-I editing site within the seed region of miR-376b-3p and demonstrated that the unedited form enhances both proliferation and differentiation of goat MuSCs, accompanied by elevated expression of proliferation and differentiation markers, such as *Pax7*, *PCNA*, *MyoD*, and *MyoG*. These findings suggest that the unedited isoform may indirectly influence gene expression by activating myogenic regulatory networks. In contrast, the edited miR-376b-3p exhibited markedly attenuated effects on these processes, indicating that RNA editing can act as a molecular switch that fine-tunes miRNA functionality during muscle development. Consistent with our results, A-to-I editing of miR-378a-3p in melanoma occurs in nonmetastatic but not metastatic cells, where the edited isoform uniquely targets the oncogene *PARVA* to suppress malignant progression, a property absent in the unedited form (44).

Editing within the miRNA seed region can reprogram miRNA–mRNA interactions, thereby modulating cellular function (42, 45). More generally, A-to-I editing can weaken existing miRNA binding, generate novel sites, or disrupt recognition entirely, as shown in 3′UTRs of *AVPR1A*, *TMX4*, and *ACTN2*, where edited transcripts exhibited altered responsiveness to miR-21, miR-378, and miR-133b, respectively (13). Similarly, editing events affect key myogenic regulators, such as *TRAF6*, *NALF1*, and *SLC38A1*, in goat MuSCs (16). These findings highlight A-to-I modifications as a dynamic mechanism for modulating post-transcriptional regulation, reconfiguring miRNA targeting, and influencing cell proliferation and differentiation. In this study, we identified *RYBP* as a direct target of unedited miR-376b-3p, which suppresses this negative myogenic regulator and consequently promotes differentiation. This is consistent with previous studies showing that *RYBP* functions as a repressor of myogenesis in both mice and cattle (35, 36). RYBP is a transcriptional coregulator and a component of the variant polycomb repressive complex 1 (PRC1), which catalyzes histone H2A monoubiquitination (H2AK119ub) to silence gene expression (46, 47). Unlike canonical PRC1, whose recruitment depends on PRC2-mediated H3K27me3 recognition by CBX proteins, variant PRC1 can bind chromatin independently of H3K27me3 *via* RYBP. The RYBP–PRC1 complex interacts with RING1B, the catalytic subunit responsible for H2AK119ub deposition, revealing an H3K27me3-independent pathway for PRC1 recruitment (48). The RYBP–YY1–Ezh2 axis maintains the silencing of myogenic promoters and miRNAs, such as miR-29 and miR-1, during proliferation, whereas its dissociation during differentiation activates muscle-specific transcriptional programs (36). RYBP loss impairs neural fate commitment and derepresses Wnt and non-neural genes, whereas Yaf2 deletion redistributes RYBP binding, elevates H2AK119ub, and promotes neural differentiation in mouse embryonic stem cells (49). Collectively, these findings identify RYBP as a dynamic chromatin regulator that bridges ubiquitination with polycomb repression, fine-tuning lineage-specific transcriptional programs. Given this, our finding that the unedited form of miR-376b-3p directly targets RYBP suggests that miRNA editing may modulate polycomb group (PcG)–mediated chromatin dynamics. Reduced RYBP expression could relieve PcG repression and facilitate transcriptional activation during muscle differentiation. Future studies integrating chromatin immunoprecipitation sequencing and epigenomic profiling will clarify whether miR-376b-3p-mediated regulation of RYBP influences PcG activity and chromatin remodeling in myogenesis.

Notably, *RYBP* overexpression had no measurable impact on MuSC proliferation in our system, indicating that the proliferative effect of miR-376b-3p is unlikely to be mediated through *RYBP*. Instead, miR-376b-3p may engage alternative targets during the proliferative phase. This notion is consistent with the individual miRNAs exerting stage-specific functions by acting on distinct downstream genes. For example, miR-10b-5p promotes myogenic differentiation by repressing *NFAT5* and may influence proliferation *via KLF4* (50); miR-664-5p drives proliferation through *SRF*, whereas inhibiting differentiation through the Wnt/β-catenin pathway (51); and miR-2425-5p regulates bovine satellite-cell proliferation and differentiation *via RAD9A* and *MYOG*, respectively (52). In our study, 24 potential targets of miR-376b-3p were computationally predicted, many of which lose binding affinity upon A-to-I editing. Among them, *GAB1* is particularly noteworthy, as its overexpression suppresses oxidized-LDL–induced proliferation and migration in vascular smooth muscle cells (53). These data indicate that *GAB1* may represent a proliferation-related target of miR-376b-3p, although definitive mechanistic validation will require targeted functional assays.

Collectively, our findings reveal that A-to-I RNA editing of miR-376b-3p functions as a crucial post-transcriptional mechanism regulating myogenic differentiation through its target *RYBP*. The unedited miR-376b-3p promotes muscle differentiation primarily by alleviating *RYBP*-mediated repression, whereas its proliferative effect may involve additional pathways independent of *RYBP*. In contrast, A-to-I editing abolishes *RYBP* targeting and attenuates the differentiation-promoting activity of miR-376b-3p, highlighting RNA editing as a fine-tuning mechanism that expands miRNA functional plasticity and balances proliferation and differentiation in muscle stem cells. These findings provide new insight into the regulatory complexity of RNA editing in skeletal muscle development and suggest that modulating RNA editing activity or miR-376b-3p editing balance may represent a potential strategy to promote muscle regeneration and repair.

## Experimental procedures

### Animals and sample collection

The 1-month-old female Chengdu gray goats (n = 3) were from the Chengdu grey goat breeding farm and were humanely sacrificed by animal welfare guidelines. Tissue samples, including heart, liver, spleen, lung, kidney, LD muscle, psoas major muscle, semitendinosus muscle, and gastrocnemius muscle, were collected immediately postmortem and rapidly frozen in liquid nitrogen for subsequent analyses. In addition, a portion of the LD muscle was used for the isolation of skeletal MuSCs.

### Cell isolation and identification

Goat skeletal MuSCs were isolated from LD muscle as previously described.^24^ Briefly, freshly harvested LD muscle was rinsed three to five times with sterile PBS (Servicebio), minced, and digested with 0.2% trypsin–EDTA (Gibco) at 37 °C for 1 h. After centrifugation at 1500*g* for 5 min, the cell pellet was resuspended in high-glucose Dulbecco's modified Eagle's medium (DMEM) (Servicebio) supplemented with 10% fetal bovine serum (Gibco). The suspension was sequentially filtered through 70 μm and 40 μm cell strainers and centrifuged at 800*g* for 5 min. MuSCs were enriched by Percoll gradient centrifugation (20%, 40%, and 90%), and cells collected from the 40%/90% interface were cultured and subsequently used for immunofluorescent staining. Pax7 (mouse anti-Pax7, 1:50 dilution; Santa Cruz), a specific marker of MuSCs, and MyHC (mouse anti-MyHC, 1:50 dilution; Santa Cruz), a marker of muscle differentiation, were used for identification.

### Cell culture and transfection

Goat MuSCs were cultured in an incubator at 37 °C with 5% CO_2_. Cells were seeded at an initial density of 2 × 10^5^ cells/ml in 6-well plates or 1 × 10^5^ cells/ml in 12-well plates and maintained in growth medium consisting of DMEM supplemented with 10% fetal bovine serum and 2% penicillin–streptomycin. Upon reaching 80% to 90% confluence, myogenic differentiation was induced using a differentiation medium composed of DMEM supplemented with 2% horse serum (Gibco).

For proliferation assays, cells at ∼50% confluence were transfected with *RYBP* overexpression plasmids (pRYBP), *ADAR1* overexpression plasmids (pADAR1), or NC vectors (pNC) (Tsingke), or with synthetic mimics of WT (miR-A), edited (miR-G) chi-miR-376b-3p, or NC miRNA (miR-NC) (RiboBio) using Lipofectamine 3000 (Invitrogen) according to the manufacturer’s instructions. For differentiation assays, transfections were performed on day 2 after differentiation medium induction using the same reagents and protocols. Cells were collected 48 h post-transfection for RNA and protein extraction, and MyHC immunofluorescence staining was conducted at 72 h to assess myogenic differentiation.

### Gene and miRNA expression quantification

Total RNA was extracted from goat tissues and cultured MuSCs using RNAiso Plus reagent (Takara) according to the manufacturer’s protocol. The concentration and purity of RNA were measured using a NanoDrop 2000 spectrophotometer (Thermo Fisher Scientific), and RNA integrity was confirmed by 1.5% agarose gel electrophoresis. For gene expression analysis, 1 μg of total RNA was reverse-transcribed into complementary DNA using the PrimeScript RT Reagent Kit with gDNA Eraser (Vazyme) for mRNA detection, or the miRNA PrimeScript RT Kit (Takara) for miRNA analysis. RT–qPCR was performed using SYBR Premix Ex Taq II reagent (Takara) on a CFX96 real-time PCR detection system. GAPDH and U6 were used as references for mRNA and miRNA normalization, respectively. Relative expression was calculated using the 2^−ΔΔCt^ method. To differentiate between the WT and edited forms of chi-miR-376b-3p, which differ by a single nucleotide, locked nucleic acid–modified primers were designed and synthesized (Tsingke) to ensure specificity (54). The sequences of the primers used are listed in Table 1.

**Table 1:** RT–qPCR primer information

Gene name	Primer sequence	
	Forward (5′-3′)	Reverse (5′-3′)
GAPDH	F: GCAAGTTCCACGGCACAG	R: GGTTCACGCCCATCACAA
PAX7	F: AGGACGAAGCGGACAAGAA	R: TCCAGACGGTTCCCTTTGT
PCNA	F: TGAAGAAAGTGCTGGAGGCG	R: TTTGGACATGCTGGTGAGG
MyoD	F: GTGCAAACGCAAGACGACTA	R: GCTGGTTTGGGTTGCTAGAC
MyoG	F: GGACCCTACAGATGCCCACAA	R: TTGGTATGGTTTCATCTGGG
MyHC	F: AGTCTTTGTGGCGGACCCTA	R:TTGGCTGTCACCTTCCGCC
RYBP	F: GAATCACACTTCAAGGCCC	R: GGATGAGGATGTCGAGGAGG
GAB1	F: CCAACTCTCCACCACGACAA	R: GGTGCTGGTTTGACTTTTCTGT
WWTR1	F: CCCGGAGTTTGGTACTGGTTT	R: CCACCCAAAGCCCTCAGTAA
SETD7	F: TTCAAAGGTAGCTGCGGGAC	R: ATCAAGGGAGAGCGTGTTCC
XPR1	F: GCTCAACGCAGGTTTGCTAC	R: AGACTGGCTTTCTGCGTTGT
PLAG1	F: GCCACTGTCATTCCTGGTGA	R: AGGCTTTCGTGCAGTCTTGT
CRISPLD1	F: GGCCCAAAGCTGTTTACCTG	R: TCCAAAACTCGGTGGGCAAG
SLC4A4	F: AGACAGGCCACCGAGAAAAG	R: TCCTCGCCCAAGATGAATCG
GPR180	F: TGTGCCATCCAGGTCTTGTG	R: AGTGAGGCCGAGTTTTGTGT
EBF2	F: CAGTCACCAAGCAGCCCATA	R: AACACCGACTGCACATCACT
U6	F: TGGAACGCTTCACGAATTTGCG	R: GGAACGATACAGAGAAGATTAGC
chi-miR-376b-3p (miR-WT)	F: GGCGATCATAGAGGAAAATCCAT	R: GTGCAGGGTCCGAGGT
chi-miR-376b-3p (miR-E)	F: GGCATCATGGAGGAAAATCCAT	R: GTGCAGGGTCCGAGGT

Abbreviations: miR-E, edited miR-376-3p; miR-WT, unedited miR-376-3p.

### Western blot

Total protein was extracted from proliferating and differentiating MuSCs using a protein extraction kit (Solarbio) and quantified using a BCA protein assay kit (BestBio) following the manufacturer’s protocols. Equal amounts of protein (20 μg per sample) were separated by SDS-PAGE and transferred onto polyvinylidene fluoride membranes (Millipore). Membranes were blocked and incubated overnight at 4 °C with the appropriate primary antibodies Pax7 (1:200 dilution; Santa Cruz), PCNA (1:1000 dilution; Abclonal), MyoG (1:1000 dilution; Bioss), MyoD (1:1000 dilution; Proteintech), MyHC (1:1000 dilution; Zen Bio), and β-tublin (1:2000 dilution; Zen Bio), followed by incubation with horseradish peroxidase–conjugated secondary antibodies (1:5000 dilution; Zen Bio) at 37 °C for 2 h. Protein signals were visualized using enhanced chemiluminescence reagents (Pierce). β-tubulin was used as a loading control.

### EdU assays

At 24 h post-transfection, cells were incubated with 10 μM EdU (RiboBio) at 37 °C for 2 h to label proliferating cells. After incubation, cells were fixed with 4% paraformaldehyde for 30 min at room temperature, permeabilized with 0.5% Triton X-100 for 10 min, and stained with the reaction reagent for 30 min. Nuclei were stained with Hoechst 33342 for 30 min. Fluorescence images were acquired using an inverted fluorescence microscope, and the number of EdU-positive nuclei was quantified using ImageJ software (National Institutes of Health). The EdU incorporation rate was calculated as the percentage of EdU-positive cells relative to total nuclei.

### Immunofluorescence assays

Cells induced to differentiate for 72 h post-transfection were fixed with 4% paraformaldehyde at room temperature for 15 min. After washing with PBS, cells were permeabilized with 0.5% Triton X-100 at 4 °C for 10 min. Following three PBS washes, cells were blocked with 2% bovine serum albumin at 37 °C for 30 min, then incubated with anti-mouse MyHC antibody (1:50 dilution; Santa Cruz) overnight at 4 °C. Subsequently, cells were incubated with secondary antibody IgG (H + L) (1:200 dilution; ABclonal) at 37 °C for 2 h. Nuclear staining was performed using 4',6-diamidino-2-phenylindole at room temperature for 10 min in the dark. Fluorescent images were captured using an inverted fluorescence microscope and analyzed with ImageJ software. The fusion index was calculated as the percentage of nuclei within MyHC-positive myotubes containing three or more nuclei relative to the total number of nuclei.

### Bioinformatics analysis

Owing to the limited availability of goat-specific databases, human miRNA target prediction databases, miRDB (https://mirdb.org/), TargetScan (https://www.targetscan.org/), miRPathDB (https://mpd.bioinf.uni-sb.de/), miRMap (https://mirmap.ezlab.org/), and miRWalk (http://mirwalk.umm.uni-heidelberg.de/), were used to predict the potential target genes of miR-376b-3p (accessed on April 10, 2023), as the seed sequence of goat miR-376b-3p is conserved with that of the human counterpart. A total of 24 overlapping genes were identified as putative targets of goat miR-376b-3p based on the intersection of results from these platforms.

### Double luciferase reporter assay

To validate the interaction between chi-miR-376b-3p and the 3′UTR of *RYBP*, WT and mutant 3′UTR fragments containing the predicted miR-376b-3p binding site were synthesized and cloned into the psiCHECK-2 luciferase reporter vector (Tsingke). Goat MuSCs were cotransfected with these reporter constructs and miR-A and miR-G mimics and cultured at 37 °C in 5% CO_2_ for 24 h. Luciferase activity was measured using the TransDetect Double-Luciferase Reporter Assay Kit (TransGen Biotech) according to the manufacturer’s instructions.

### Statistical analysis

All data were presented as mean ± standard deviation from at least three independent biological replicates. Statistical analyses were conducted using GraphPad Prism 8.0 (GraphPad Software). Comparisons between two groups were performed using an unpaired two-tailed Student’s *t* test, whereas one-way ANOVA was applied for comparisons among multiple groups. A *p* value <0.05 was considered statistically significant. Significance levels are indicated as follows: ns, not significant; *p* < 0.05 (∗); *p* < 0.01 (∗∗).

## Ethics approval

All experiments in this study were conducted strictly according to the Regulations on the Administration of Laboratory Animal Affairs issued by the Ministry of Science and Technology of China and approved by the Committee of Animal Care and Use of Sichuan Agricultural University (SAU-20221725).

## Data availability

All data are included within this article.

## Conflict of interest

The authors declare that they have no conflicts of interest with the contents of this article.
